# Amyloid goiter: A Tunisian case

**DOI:** 10.12688/f1000research.162724.1

**Published:** 2025-03-25

**Authors:** Rihab Laamouri, Ibtissem Ben Nacef, Makram Tbini, Sabrine Mekni, Yosra Htira, Sawssen Essayeh, Imen Rojbi

**Affiliations:** 1 University of Tunis El Manar Faculty of Medicine of Tunis, Tunis, Tunis, Tunisia; 2 Endocrinology, Charles Nicolle Hospital, Tunis, Tunis, Tunisia; 3 Laboratory of Renal Pathology LR00SP01, Charles Nicolle Hospital, Tunis, Tunis, Tunisia; 4 Ear Nose Throat department, Charles Nicolle Hospital, Tunis, Tunis, Tunisia

**Keywords:** Amyloid goiter, fine needle aspiration, thyroidectomy, pathological examination

## Abstract

**Introduction:**

Amyloidosis is a systemic disease caused by amyloid chain deposition. The thyroid is a very uncommon location. The prevalence of amyloid goiter is estimated to be 0.17.

**Cases presentation:**

We report three cases of amyloid goiter (AG). Three men, aged between 30 and 42 years, were hospitalized in the medical department. Compressive signs were present in one patient. All the patients had normal thyroid hormone levels. Fine Needle Aspiration (FNA) revealed amyloid deposition in one case. All the patients underwent total thyroidectomy. All patients underwent surgery without complications and were treated with L thyroxine replacement therapy. A pathological examination confirmed the diagnosis of AG. A literature review was conducted using PubMed from 2019 to 2024.

**Conclusion:**

Amyloid deposition is rarely associated with thyroid dysfunction. The discovery circumstance is generally thyroid enlargement and may be associated with compressive signs. Compressive and rapid enlargement of the thyroid may be an indication for surgery. FNA is generally less informative than core biopsy and surgery . Thyroidectomy is a therapeutic and diagnostic procedure. Clinicians should consider amyloid deposition in front of a growing goiter, particularly when systemic amyloidosis is known.

## Introduction

Amyloidosis is a systemic disorder characterized by extracellular deposition of amyloid fibrillary proteins. ^1^ This is an aggregation of more than 20 proteins belonging to families that do not exhibit structural or functional relationships. It can affect any organ and can manifest in a localized form or as a generalized disease. The mechanisms underlying its pathogenesis are poorly understood.

Pathological studies can identify this deposition by its affinity to the Congo Red stain and yellow-green birefringence under polarized light. ^2^ Several types of amyloidosis exist, which are distinguished by the type of protein and the localization of the deposit.

A histological study of amyloid deposition in the thyroid gland revealed its presence in 30%–80% of cases. ^3^ However, the prevalence of amyloid goiter in multinodular goiters remains low with an estimated prevalence of 0.17. ^4^ The potential to simulate thyroid cancer by its volume and progression is also a concern. ^5^ The earliest documented case of amyloid goiter (AG) was reported by Beckman in 1858. ^6^ This article presents a comprehensive review of three cases of AG, complemented by a thorough literature review. This study aimed to elucidate the clinical, therapeutic, and evolutionary characteristics of this rare entity.

## Case report

### Case 1

A 30-year-old patient with a medical history of bilateral bronchial dilation of unknown etiology presented to the endocrinology department for the evaluation of a compressive goiter that had been present for three years. The patient reported a significant weight loss. A thorough clinical examination revealed a heterogeneous goiter that was voluminous, avascular, and hard in consistency, accompanied by multiple nodules. Thyroid assessment (thyroid-stimulating hormone and free T4) was normal. Cervical ultrasound revealed a multinodular goiter with the largest thyroid nodule measuring 6 cm. No cervical lymphadenopathy was detected. Anti-thyroid antibodies (ATAb) and Koch bacillus tests were negative, and surgical intervention was deemed necessary because of compressive symptoms and suspicious nodules. The patient underwent total thyroidectomy, and a pathological study revealed AG. A labial biopsy with Congo red coloration was later performed, confirming the diagnosis of amyloidosis AA secondary to bronchial dilation. The patient was then placed in a Colchicine, and low blood pressure were observed during monitoring. Adrenal insufficiency was suspected and confirmed using a Synachtene stimulation test, with a peak cortisol level of 457 nmol/L (550 nmol/L). The patient was prescribed hydrocortisone. The diagnosis was peripheral adrenal insufficiency, likely due to amyloid deposition in the adrenal glands, which is related to systemic amyloidosis.

### Case 2

A 45-year-old patient with ankyloarthropathy was admitted to our hospital with a diagnosis of chronic glomerular nephropathy, as indicated by lower limb edema and pure nephrotic syndrome. Upon clinical examination, the patient exhibited macroglossia and diminished osteotendinous reflexes. Renal biopsy confirmed the diagnosis of renal amyloidosis. The patient was prescribed colchicine (1 mg/day). Thyroid examination revealed a multinodular goiter, as confirmed by subsequent cervical ultrasound. No compressive signs were identified during clinical examination or ultrasound imaging. The patient exhibited no clinical signs of thyroid dysfunction, and the biochemical assessment of the thyroid gland was within normal limits. Ultrasound-guided fine-needle aspiration (FNA) was performed, which confirmed the presence of amyloidosis. The patient underwent total thyroidectomy and was treated with l-thyroxine replacement therapy.

### Case 3

**Figure 1.**
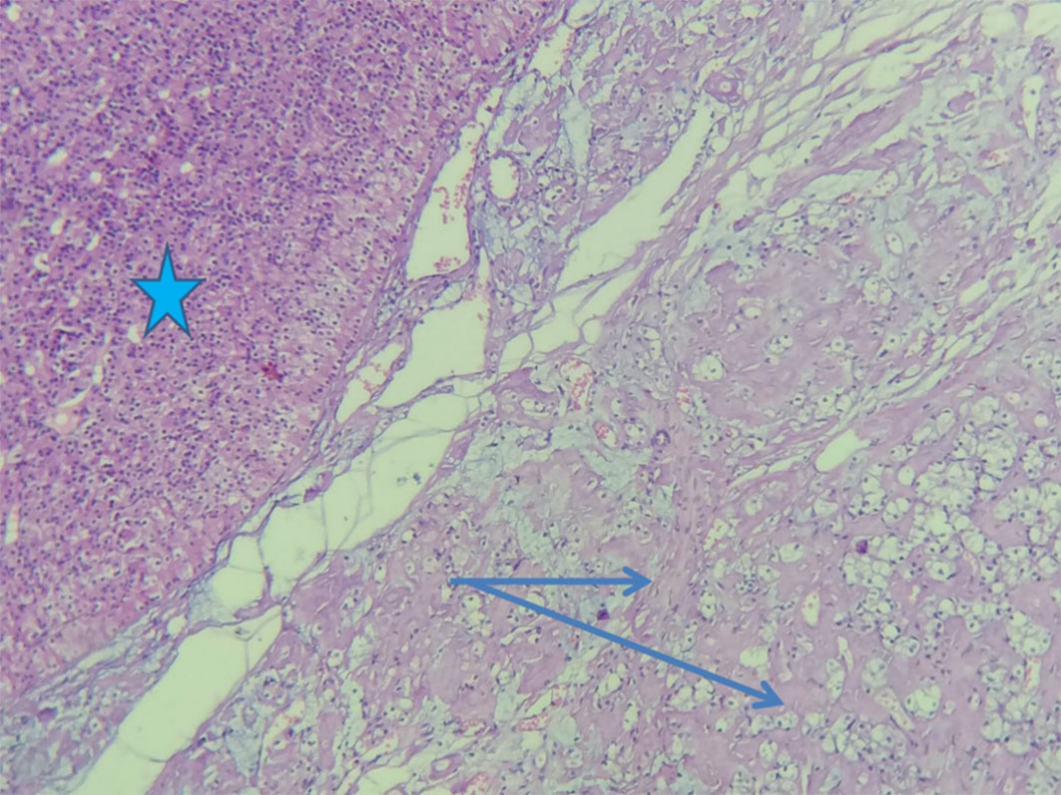
Histological study of thyroidectomy speciemen: Hematoxylin Eosine stain (Case number 3). Anhistic, finely cracked amyloid deposit (arrows) within the parathyroid parenchyma (star).

A 32-year-old patient with a medical history of chronic osteitis complicated by repeated skin infections was admitted to the hospital with impure nephrotic syndrome, which was associated with high blood pressure, hematuria, and renal failure. Biopsy of the accessory salivary glands was performed, confirming the diagnosis of amyloidosis. Subsequently, the patient developed a goiter with endothoracic extension without thyroid dysfunction or compressive signs. The thyroid assessment was normal, and ATAb was negative for anti-ATAb. The patient underwent a cervicothoracic CT scan, which showed a goiter extending beyond the upper cervical canal (18 mm to the right and left), without compressive signs. FNA revealed the presence of a few colloidal clusters. Total thyroidectomy was performed, and pathological examination revealed histological features of an amyloid goiter. Intraoperative investigations revealed a parathyroid nodule that was surgically removed. The histological study indicated parathyroid localization of amyloidosis. Additionally, the phosphocalcic assessment was within normal limits prior to surgery ( Figure 1).

## Discussion

Amyloidosis is characterized by the deposition of amyloid, an insoluble, beta-pleated sheet protein. It can occur because of chronic inflammation in cases of secondary amyloidosis, which may be associated with rheumatoid arthritis, ankylosing spondylitis, and Familial Mediterranean Fever (FMF). In the present case series, three patients had a known chronic inflammatory disease. Conversely, amyloidosis can manifest as a primary condition without an identified underlying disease. The occurrence of amyloid goiter is rare, with case reports in the literature primarily focusing on adults between the ages of 40 years and 50. However, there have been reports of cases in children and elderly individuals, indicating no clear predilection for age or sex. Amyloidosis is most often a secondary complication of an underlying inflammatory disease. ^7, 8^

Progressive enlargement of the thyroid was observed clinically, and compressive signs have been documented in numerous cases in the literature. In our case, this was reported in one patient and was the primary symptom that motivated her to consult. This rapid enlargement may be attributed to the inflammation of the thyroid gland. In most cases, surgical intervention is performed to exclude malignancy. Amyloid deposition is rarely associated with thyroid dysfunctions. Our three patients exhibited clinical and biological euthyroidism, and the literature on the subject reported only a few cases of thyroid dysfunction. ^9^

FNA has been demonstrated to be a valuable diagnostic tool in several cases; however, it does not appear to be a reliable indicator of amyloidosis. In our experience, FNA was performed in two cases, and amyloid deposition was identified on cytological examination in one. Nonetheless, as reported in some cases in the literature, core biopsy was more informative and amyloid deposition was revealed when it was performed. Definitive histological examinations remain the gold standard for diagnosis. Thyroidectomy serves a dual role as both a therapeutic and a diagnostic modality. Notably, amyloid deposition has the potential to affect other endocrine systems. As detailed in this report, one patient exhibited parathyroid amyloid deposits, which were substantiated through pathological examination despite normal calcium and phosphorus levels in the assessment. Adrenal amyloid deposition was suspected in another patient who presented with primary adrenal insufficiency. Patel et al. have also documented parathyroid amyloid deposits. ^8^

The patient with amyloidosis secondary to FMF was diagnosed with adrenal insufficiency. We hypothesized that the patient's adrenal amyloid deposition or a condition associated with FMF was the underlying cause. To expand on this finding, a comprehensive literature review was conducted using the term "amyloid goiter" in PubMed. The search was limited to publications published between 2019 and 2024. The results of this review are summarized in Table 1.

**Table 1.** Characteristics of patients with amyloid goiter reported in the literature.

	Cases/Year	Age	Gender	Clinical features	Thyroid test	US	FNA/Core biopsy	Etiology of amyloidosis	Treatment	Pathological examination
1	Gonzalez et al 2024 ^10^	64	F	Thyroid enlargement	Hyperthyroidism	Goiter	Core Biopsy: amyloid deposit	-	Anti thyroid medication	-
2	Larenjeira et al 2024 ^11^	75	F	Dysphagia	Euthyroid	Goiter Nodules 11mm	FNA: benign cyst and follicular lesion of undetermined significance	Heavy chain amyloidosis	Chemotherapy	-
3	Denizmen et al 2024 ^12^						Biopsy: Amyloid substance			
4	George et al 2024 ^13^	51	F	Thyroid enlargement	Hyperthyroidism	-	-	Primary amyloidosis	Total thyroidectomy	Amorphous material Papillary carcinoma
5	Gokbulut et al 2024 ^14^	26	F	Neck pain	Euthyroid	-	Core biopsy: Amyloid deposit	FMF	-	-
6	Khan et al 2023 ^15^	38	F	Hoerseness	Euthyroid	-	FNA: Bethesda II	-	Total thyroidectomy	Eosinophilic amorphous substance
7	Khader et al 2023 ^16^	42	M	Thyroid enlargement	Hyperthyroidism	Heterogenous goiter, no nodules	FNA: no malignancy signs	Behçet disease	Total thyroidectomy	Eosinophilic amorphous fibrillary material+ fatty metaplasia
8	Moreno et al 2023 ^17^	21	F	Compressive signs	Euthyroid	Diffuse thyroid enlargement	FNA: fibro inlammatory lesions	FMF	Total thyroidectomy	Amyloid deposition
9	Chihiro et al 2022 ^18^	60	F	Autopsy	-	-	-	Rhymatoid arthritis	-	Eosinophilic amorphous deposits with parenchymal cell destruction
10	Patel et al 2022 ^8^	73	M	Dysphagia Hoerseness	Euthyroid	Goiter Nodules	FNA: Bethesda I	Multiple myeloma	Total thyroidectomy	Amyloid deposition
11	Morado da silva et al 2022 ^19^	54	F	Dysphagia Hoerseness	Euthyroid	Goiter Nodule 28mm	FNA: Bethesda IV	chronic pyelonephritis and bronchiectasis	Total thyroidectomy	Papillary carcinoma Eosnophilic amprphous material
12	Chincholi et al 2022 ^20^	36	F	Thyroid enlargement Dysphagia	Euthyroid	Goiter Nodules	FNA: Bethesda II	Localized AA amyloidosis	Total thyroidectomy	Eosinophilic amorphous material
13	Cavaco et al 2021 ^21^	46	M	Thyroid enlargement	Euthyroid	Goiter Nodules	follicular lesion of undetermined significance	-	Total thyroidectomy	Eosinophilic amorphous material
14	Ledesma et al 2021 ^22^	66	M	Autopsy	Euthyroid	-	-	Primary amyloidosis	-	Amyloid deposition
15	Şeker et al 2020 ^23^	55	M	Dysphagia	Euthyroid	Goiter Nodules: the largest 50mm	FNA: benign thyroid cytology	Rhumatoid arthritis	Total Thyroidectomy	Amyloid desposition
16	Lari et al 2020 ^24^	53	M	Thyroid enlargement	Euthyroid	Homogenous goiter	FNA: benign cytology	Primary Amyloidosis	Total thyroidectomy	Eosinophilic amorphous deposition
17	Jacubovic et al 2020 ^25^	40	M	Dyspnea Hoarseness	Euthyroid	Enlargement gland, cystic nodules	FNA: non diagnostic Biopsy: amyloid deposits	Osteomyelitis	Total thyroidectomy	Eosinophilic amorphous material
18	Abukhalaf et al 2020 ^26^	23	M	Thyroid enlargement	Euthyroid	Enlargement gland	FNA: suggestive amyloidosis	FMF	Colchicine	-
19	Orrego et al 2019 ^27^	70	M	Thyroid enlargement	Euthyroid	Goiter, Nodules the largest 39mm	FNA: Bethesda III	Primary amyloidosis	Lobectomy	Amyloid deposition
20	Lopez et al 2019 ^28^	48	F	Dysphagia	Subclinical hyperthyroidism	Enlargement gland	Core biopsy: eosinophilic amyloid deposit	Rhumatoid arthritis	Total thyroidectomy	Amyloid deposition

US: ultrasonography; FNA: Fine Needle Aspiration; FMF: Familial Mediterranean Fever.

## Ethics and consent

Ethical approval and consent were not required.

## Consent to published

Written informed consent for the publication of the report and associated images was obtained from the patients prior to submission.

## Data Availability

No data are associated with this article.
