# Supplementary material for: Amyloid goiter: A Tunisian case series
Source: F1000Res. 2025 Oct 20;14:324. Originally published 2025 Mar 25. [Version 2] doi: 10.12688/f1000research.162724.2 (PMC12739365; doi:10.12688/f1000research.162724.2)
Supplement: Peer review history

# Reviewer report: Reviewer response for version 2

**DOI:** 10.5256/f1000research.189864.r425640

**Published:** 2025-12-24

**Authors:** José L. Paz-Ibarra

**Affiliations:** 1 Universidad Nacional Mayor de San Marcos, Lima, Peru

I respectfully offer the following comments to strengthen the clarity, scientific impact, and academic rigor of your work.

 Expand the differential diagnosis reasoning

 To enhance clinical relevance, consider discussing:

 What competing diagnoses were considered early in the evaluation,

 Which clinical, biochemical, or histopathologic clues shifted the diagnostic direction,

 Why certain alternative diagnoses were excluded.

 This level of detail mirrors best practices in reporting rare endocrine disorders.

 Provide more depth regarding pathology and immunohistochemistry

 Drawing from experience with amyloid goiter—where Congo red staining and detailed histopathologic architecture were crucial—the pathology section here would benefit from:

 clearer description of staining patterns,

 structured analysis of histopathologic criteria,

 discussion of any borderline or ambiguous findings,

 relevance of each immunomarker in differentiating this condition from look-alikes.

 Such reinforcement helps non-pathologists better understand the diagnostic basis.

 Improve the manuscript’s structural cohesion

 Several paragraphs would benefit from tighter transitions, clearer section boundaries, and reduction of redundancy.

 A reorganized flow—introduction → diagnostic pathway → pathology → management → implications—would improve readability.

 The summary table of the reviewed literature does not include the patient cases from the references used

Are enough details provided of any physical examination and diagnostic tests, treatment given and outcomes?

Partly

Is the background of the cases’ history and progression described in sufficient detail?

Yes

Is sufficient discussion included of the importance of the findings and their relevance to future understanding of disease processes, diagnosis or treatment?

Yes

Is the conclusion balanced and justified on the basis of the findings?

Partly

Reviewer Expertise:

Thyroid diseases

I confirm that I have read this submission and believe that I have an appropriate level of expertise to confirm that it is of an acceptable scientific standard, however I have significant reservations, as outlined above.

# Reviewer report: Reviewer response for version 2

**DOI:** 10.5256/f1000research.189864.r430137

**Published:** 2025-12-08

**Authors:** Maria M Picken

**Affiliations:** 1 Loyola University Chicago, Chicago, USA

Laamouri et al describe 3 patients with amyloid goiter and provide a literature review on this subject.  This is a revised version and the reviewer has a few suggestions for further improvement as follows:

 In the introduction: “Amyloidosis is a systemic disease caused by amyloid chain deposition deposition of misfolded proteins. 

 Case 1:  bronchial dilation – please replace with bronchiectasis

 Case 2: chronic glomerular nephropathy – please replace with nephropathy

 “Renal biopsy confirmed the diagnosis of renal amyloidosis. The patient was prescribed colchicine (1 mg/day).”  Please add that based on the kidney biopsy and clinical studies, a presumptive diagnosis of AA amyloidosis was made.

 Case 3:” impure” nephrotic syndrome? Please remove “impure”

 “Anhistic, finely cracked” – please replace with “acellular homogeneous” deposits (arrows) within the parathyroid parenchyma (star).  These deposits displayed yellow-green birefringence with Congo red stain diagnostic of amyloid (not shown).

 Discussion:

 “Conversely, amyloidosis can manifest as a primary condition without an identified underlying disease” – please rephrase:

 In other cases, amyloidosis can seemingly manifest as a primary condition but upon further investigations underlying plasma cell dyscrasia can be detected in most patients

Are enough details provided of any physical examination and diagnostic tests, treatment given and outcomes?

Yes

Is the background of the cases’ history and progression described in sufficient detail?

Partly

Is sufficient discussion included of the importance of the findings and their relevance to future understanding of disease processes, diagnosis or treatment?

Yes

Is the conclusion balanced and justified on the basis of the findings?

Yes

Reviewer Expertise:

NA

I confirm that I have read this submission and believe that I have an appropriate level of expertise to confirm that it is of an acceptable scientific standard, however I have significant reservations, as outlined above.

# Reviewer report: Reviewer response for version 2

**DOI:** 10.5256/f1000research.189864.r425634

**Published:** 2025-11-12

**Authors:** Najla Bchir

**Affiliations:** 1 Hôpital militaire de Tunis, Tunis, Tunisia

**1. Summary of the case**

 This report presents three male patients with AG; one had compressive symptoms. All had normal thyroid function. Fine Needle Aspiration identified amyloid in only one case. All patients underwent total thyroidectomy and received L-thyroxine therapy. Pathology confirmed AG. Literature review (2019–2024) showed that AG often presents as thyroid enlargement, sometimes with compressive signs and thyroid dysfunction is uncommon. Surgery serves both diagnostic and therapeutic roles, while FNA is generally less informative. Clinicians should consider amyloid deposition in growing goiters, especially in patients with systemic amyloidosis.

**2. General assessment**

 The case is well-presented and reports a rare but clinically relevant condition. The diagnostic and therapeutic approaches are clearly described. However, the discussion could benefit from a deeper comparison with existing literature.

**3. Strengths**

- Clear chronological presentation of clinical events.
- clear and well-structured conclusio: It appropriately highlights the rarity of thyroid amyloid deposition and emphasizes the key clinical features and management considerations.

**4. Major comments**

 -Detail the prognostic aspect:Given the clinical and ultrasound features suggestive of malignancy, discuss the possible association with thyroid cancers, particularly since this relationship has been reported in the literature

**5. Minor comments**

- Please correct “prevalence of amyloid goiter is estimated to be 0.17.” to “prevalence of amyloid goiter is estimated at 0.17%.
- Please correct “Thyroid assessment” to “hormonal thyroid assessment“
- Please correct “amyloidosis AA secondary to bronchial dilation“ to “amyloidosis AG“.”

**6. Novelty and educational value** *:*

 Although similar cases have been reported, this article is notable for the multidisciplinary management approach, providing an important teaching point.

**7. Data and ethical compliance**

 The authors state that informed consent was obtained from the patients, and ethical approval was not required for these case reports. This complies with F1000Research policy.

Are enough details provided of any physical examination and diagnostic tests, treatment given and outcomes?

Yes

Is the background of the cases’ history and progression described in sufficient detail?

Yes

Is sufficient discussion included of the importance of the findings and their relevance to future understanding of disease processes, diagnosis or treatment?

Partly

Is the conclusion balanced and justified on the basis of the findings?

Yes

Reviewer Expertise:

endocrine disorders

I confirm that I have read this submission and believe that I have an appropriate level of expertise to confirm that it is of an acceptable scientific standard.
